# “When I saw walking I just kind of took it as wheeling”: interpretations of mobility-related items in generic, preference-based health state instruments in the context of spinal cord injury

**DOI:** 10.1186/s12955-016-0565-9

**Published:** 2016-11-28

**Authors:** Yvonne Anne Michel, Lidia Engel, Kim Rand-Hendriksen, Liv Ariane Augestad, David GT Whitehurst

**Affiliations:** 1 Department of Health Management and Health Economics, Institute of Health and Society, Medical Faculty, University of Oslo, Postboks 1089, Blindern, 0318 Oslo, Norway; 2 Faculty of Health Sciences, Simon Fraser University, Burnaby, BC Canada; 3 Centre for Clinical Epidemiology and Evaluation, Vancouver Coastal Health Research Institute, Vancouver, BC Canada; 4 Health Services Research Centre, Akershus University Hospital, Lørenskog, Norway; 5 International Collaboration on Repair Discoveries (ICORD), Faculty of Medicine, University of British Columbia, Vancouver, BC Canada

**Keywords:** Preference-based health state instruments, Validity, HRQoL, Mobility, Spinal cord injury, Reframing, Item interpretation

## Abstract

**Background:**

In health economic analyses, health states are typically valued using instruments with few items per dimension. Due to the generic (and often reductionist) nature of such instruments, certain groups of respondents may experience challenges in describing their health state. This study is concerned with generic, preference-based health state instruments that provide information for decisions about the allocation of resources in health care. Unlike physical measurement instruments, preference-based health state instruments provide health state values that are dependent on how respondents interpret the items. This study investigates how individuals with spinal cord injury (SCI) interpret mobility-related items contained within six preference-based health state instruments.

**Methods:**

Secondary analysis of focus group transcripts originally collected in Vancouver, Canada, explored individuals’ perceptions and interpretations of mobility-related items contained within the 15D, Assessment of Quality of Life 8-dimension (AQoL-8D), EQ-5D-5L, Health Utilities Index (HUI), Quality of Well-Being Scale Self-Administered (QWB-SA), and the 36-item Short Form health survey version 2 (SF-36v2). Ritchie and Spencer’s ‘Framework Approach’ was used to perform thematic analysis that focused on participants’ comments concerning the mobility-related items only.

**Results:**

Fifteen individuals participated in three focus groups (five per focus group). Four themes emerged: wording of mobility (e.g., ‘getting around’ vs ‘walking’), reference to aids and appliances, lack of suitable response options, and reframing of items (e.g., replacing ‘walking’ with ‘wheeling’). These themes reflected item features that respondents perceived as relevant in enabling them to describe their mobility, and response strategies that respondents could use when faced with inaccessible items.

**Conclusion:**

Investigating perceptions to mobility-related items within the context of SCI highlights substantial variation in item interpretation across six preference-based health state instruments. Studying respondents’ interpretations of items can help to understand discrepancies in the health state descriptions and values obtained from different instruments. This line of research warrants closer attention in the health economics and quality of life literature.

## Background

Generic, preference-based health state instruments play an important role in informing priorities regarding available treatment options [1]. Preference-based health state instruments consist of two components: a ‘descriptive system’ that asks respondents to describe their health by responding to questions in a standardized questionnaire (resulting in health state *descriptions*) and a ‘valuation system’ that provides preference weights (also known as index scores) that represent the values that respondents, usually from the general population, attach to living in each health state (health state *values*).

Instruments used to measure health-related quality of life (HRQoL) can be classified as either generic or specific to a particular disease, condition, or population otherwise defined (e.g., older adults). Disease-specific instruments are more sensitive to small changes in HRQoL for the target group because they focus on aspects that are known to be relevant to that group. In contrast, generic instruments are intended to be applicable to a wide range of conditions [2]. Information gained by using generic and disease-specific instruments is valuable when conducting economic evaluation to inform decision processes concerning competing demands for scarce health care resources [1, 3–6]. However, only generic instruments allow for comparisons of interventions across different diseases and patient groups.

Instruments used to measure preference-based HRQoL are often referred to as utility instruments, health state utility instruments, preference-based HRQoL instruments or health state instruments. The variety of terms used suggests an underlying lack of conceptual clarity of the definition and theory of ‘quality of life’ [7]. In this study, generic preference-based health state instruments are referred to as ‘health state instruments’. The items and dimensions contained within health state instruments differ in their conceptual coverage of physical, social and mental health. Throughout this paper, ‘item’ is referred to as “*a linguistic statement generally consisting of a stem plus a number of ordered response levels*” [8], whereas ‘dimension’ is used to describe the conceptual idea that is represented by one or several items, e.g., ‘mobility’ or ‘usual activities’ [8].

What most health state instruments have in common is that a small number of items (often only one) represent each dimension. This is in contrast to classic measurement theory in psychometrics, which recommends measuring each dimension by several items in order to improve reliability and validity [9]. Asking respondents to describe their health by choosing response options in a questionnaire is considerably different from measuring physical parameters like a person’s body temperature. While a clinical thermometer directly transforms body temperature to a displayed number, the application of a questionnaire includes at least two phases of interpretation. Firstly, the respondent needs to interpret the questions in the questionnaire in order to choose a response option. Secondly, the recipient of the data needs to interpret the results. Knowing how respondents interpret items is crucial to ensure that an instrument measures what it is supposed to measure [10]. Diverging interpretations can occur if (i) respondents interpret items in a way that diverges from the interpretation originally intended by the instrument developer, or (ii) if the item interpretation by a subgroup of respondents (e.g., wheelchair users) diverges systematically from interpretations made by the individuals outside of that subgroup. Either situation is problematic for any questionnaire that is used to capture health status (or, more broadly, quality of life) because the validity of the data is compromised. There is a sizeable literature on variation of health state values between different groups (e.g., age groups, educational groups, countries, and patients vs. general population) [11, 12], and variation in health state values observed when the same individuals describe their health using different health state instruments [8, 13–16]. Systematic differences in item interpretation across subgroups may well be a factor in explaining observed variation in health state descriptions and values.

Although the instruments used to measure HRQoL are intended for quantitative use, qualitative research is a highly appropriate means of exploring what is driving the differences in health state descriptions and values [17, 18]. As health state instruments are often used to capture impairments, it is problematic if groups that have significant levels of impairment cannot adequately describe their health state. A focus group study by Whitehurst and colleagues found that individuals living with spinal cord injury (SCI) is a subgroup that faces challenges in describing their health state [19]. These findings supported other studies–outside the context of preference-based instruments–that suggest mobility-related items in standardized, generic HRQoL questionnaires pose challenges for individuals living with SCI [20–22]. Item interpretation has been widely studied in the field of psychometrics and the need for respondents to interpret an item before they can choose an appropriate response option is a well-established phase within theoretical models that describe the response process [11, 23–25]. However, there have been relatively few studies focusing on health state instruments [10, 26]. The presence of diverging item interpretations and its impact on the validity of health state descriptions and values is an under-researched empirical question in health economics. The aim of this study is to further explore and describe difficulties faced by respondents living with SCI when answering mobility-related items included in health state instruments.

## Methods

### Data, Participants and Instruments

This study comprises secondary analysis of focus group transcript data collected in Vancouver, Canada in 2012. The aim of the original data collection was to explore the perceptions of individuals living with SCI towards all items included in health state instruments and to identify ‘preferred’ instruments from the participants’ perspective [19]. Participants were recruited from the Vancouver General Hospital Spine Program. Purposive sampling was used to include a range of participants with regard to gender, type and severity of injury, and time since injury. Consenting participants received copies of the descriptive systems of six health state instruments (further details provided below) via mail in advance of the focus group session. Participants were asked to review the descriptive systems at home, prior to attending the focus group. Based on their availability, participants were allocated into three focus groups sessions led by an experienced focus group facilitator. During the two-hour focus group sessions, participants discussed their general perceptions of the descriptive systems in line with a structured template including the following questions: (i) What are your immediate thoughts about questionnaire X? (ii) Do you feel that questionnaire X (or particular items within questionnaire X) applies to you? (iii) Do you have any further thoughts regarding questionnaire X? (iv) Given the objective of these questionnaires, is questionnaire X acceptable to you as a whole? [19]. At the beginning of each focus group, participants were informed about the objectives of the session and the role of generic health state instruments (i.e., that the instruments were designed to be applicable for all individuals rather than a specific clinical context). Full details of the sample characteristics, exclusion criteria, recruitment procedure, and conduct of the focus groups are described elsewhere [19].

Mobility-related items feature in all of the health state instruments included in this study, namely 15D [27], Assessment of Quality of Life 8-dimension (AQoL-8D) [28], EQ-5D-5L [29], Health Utilities Index (HUI) [30], Quality of Well-Being Scale Self-Administered (QWB-SA) [31], and the SF-6D (participants were asked to consider and discuss the full 36-item Short Form health survey version 2 (SF-36v2) rather than the 11 items that comprise the SF-6D [32]). However, the way the descriptive systems require respondents to describe aspects of their mobility differs markedly. This study focused on perceptions of items related to issues of mobility, physical functioning, or ambulation. Table 1 provides an overview of the mobility-related items included in the descriptive systems of the instruments, while Appendix 1 includes the exact wording of the mobility-related items.

**Table 1:** Comparison of mobility-related aspects of six generic, preference-based health state instruments ^a^

	15D	AQoL-8D	EQ-5D-5L	HUI-3	QWB-SA	SF-6D (SF-36v2)^b^
Number of items	1 of 15	2 of 35^c^	1 of 5	1 of 15	9 of (at least) 71	SF-6D: 3 of 11 SF-36v2: 10 of 36
Name of dimension	Mobility	Independent living	Mobility	Ambulation	Physical functioning	Physical functioning
Number of response options per item	5	6	5	6	4	3
Mention of aids and/or appliances	Yes	Yes	No	Yes	Yes	No
Mention of human assistance	Yes	Yes	No	Yes	Yes	No
Time frame reference	Present health status	Past week	Today	Past week	Last three days	A typical day

^a^ The specific wording of items and response options is provided in Appendix 1

^b^ Focus group participants were required to consider the 36-item instrument in its entirety, as developers of the SF-6D do not recommended using only the 11 items of the SF-36v2 that comprise the SF-6D

^c^ Two items were considered to be ‘mobility-related’; item #15 (“mobility”) and item #3 (“getting around”). This follows the approach used by Whitehurst and colleagues [16]

### Thematic analysis of focus group data

Thematic analysis of the transcript data had the explorative objective to describe how individuals living with SCI perceive and interpret mobility-related items of the six health state instruments. The analysis was guided by Ritchie and Spencer’s ‘Framework Approach’ [33]. The approach consists of several phases: researchers (a) familiarize themselves with the data, (b) identify an initial thematic framework, (c) index and sort the data, (d) review and redefine extracted data, build categories that comprise themes and, (e) interpret identified categories. Two authors (YAM, LE) independently familiarized themselves with the focus group transcripts and agreed on an initial thematic framework covering mobility-related content (phases a-c). Indexing and sorting resulted in a matrix table that summarized the data along the themes (rows) and the respective instruments (columns). Transcripts were re-read and the initial coding framework was adjusted to ensure that all relevant aspects mentioned in the focus groups were covered by the themes (phase d). To ease interpretation, themes were assigned to higher-order categories (phase e). The same two authors (YAM, LE) conducted the analysis; all members of the study team discussed the results and interpretation of each phase.

## Results

Fifteen individuals participated in three focus groups (five per focus group). Four themes emerged from the analysis (see Table 2): (i) wording of mobility, (ii) reference to aids and appliances, (iii) lack of suitable response options, and (iv) reframing of items. The first three themes describe item features that cause difficulties when respondents attempt to describe their health state; the fourth theme summarizes comments about a response strategy respondents proposed when facing inaccessible items.

**Table 2:** Description of categories and themes revealed from the thematic analysis

Category	Theme	Description
Significant features of mobility-related items	Wording of mobility	Comments regarding the text used to describe physical functioning in the instruments.
	Reference to aids and appliances	Comments referring to walking equipment and other aids (including wheelchairs).
	Lack of suitable response options	Comments concerning difficulties identifying response options that are applicable to the respondent’s condition.
Response strategies to mobility-related items	Reframing of items	Comments indicating that respondents changed the phrasing of the items during the process of interpretation.

Results are presented by theme rather than by instrument because the primary interest of this study is to describe and highlight participants’ general perceptions to mobility-related items (rather than draw comparisons across instruments). Additional information is reported in Appendix 2, which provides a systematized overview of the mobility-related content from the focus group transcripts (i.e., the themes of the final coding framework and the respective mobility-related quotes). Focus group abbreviations (i.e., FG1, FG2 and FG3) and instrument names indicate the respective instrument and focus group transcript for each quote. For example, ‘(FG2, 15D)’ indicates that the quote is in reference to the 15D, made during the second focus group.

### Wording of mobility

Participants in the focus groups commented on items that related mobility solely to walking: *“I didn’t like the choice of words…, I don’t think walking is your only parameter for mobility”* (FG1, 15D). Furthermore, participants highlighted that individuals with SCI can be mobile despite not being able to walk: *“You can still get around but you just can’t do it by walking”* (FG2, EQ-5D-5L). Most participants preferred ‘getting around’, as used in the AQoL-8D. Such wording was considered to be more inclusive: *“I liked the wording…, it doesn't exclude anybody getting around"* (FG3, AQoL-8D); *"It doesn't say anything about walking…, it just says mobility”* (FG2, AQoL-8D).

### Reference to aids and appliances

The way aids and appliances are incorporated in the items was often discussed by participants. “*They’re beating around the bush about equipment but they’re not mentioning you’re unable to walk but are you able to get around. It’s a mobility thing,…[It needs to be] defined a little bit more, if you’re trying to find wheelchair people”* (FG2, HUI). Participants regarded the manner in which equipment was mentioned in the AQoL-8D positively: *"I liked number fifteen when it talks [about] mobility, it also included aids or equipment such as wheelchairs, frames, stations and stuff because if… I have the equipment I feel comfortable with my mobility"* (FG1, AQol-8D). Participants also felt that instruments were more inclusive if the use of a wheelchair was mentioned explicitly: *“… if the wheelchair is in there…, I’d feel more part of it”* (FG1, general remark). Regarding the HUI, the combination of aids and human assistance was perceived as confusing: “*If I’m in a standing frame or a walker I can walk, but I can't walk unassisted…, none of them really helped me answer that one"* (FG1, HUI).

### Lack of suitable response options

Participants expressed difficulties in finding response options that allowed them to describe their level of physical functioning. Often these difficulties were related to narrow wording that equates mobility to walking, or to the omission of mobility aids: *“I thought that question was kind of hard to fill out. …Well it didn’t really have an option for me* per se *because like there again this question is put in front of somebody like they don’t know whether you’re in a wheelchair or you’re a quadriplegic, paraplegic or if you’re bedridden or you can walk with a cane, right?”* (FG3, 15D). All participants were wheelchair users and, unsurprisingly, this aid was referred to most often: *“I mean they’re not applicable to people who are confined on a wheelchair”* (FG1, QWB-SA). Participants had concerns about giving a false idea of their health state as a consequence of the lack of applicable and relevant response options: *“By having questions like this that have nothing to do with me, so they’re not going to get any information, or they may even get a false sense of my health status by asking questions like that”* (FG3, SF-36v2). The lack of relevant response options led some participants to choose at random: “*It is just like just close our eyes and pick one”* (FG3, 15D). However, participants did find relevant response options for the AQoL-8D: *“The question seems appropriate, people like this question because the options are relevant as opposed to walking”* (FG3, AQoL-8D).

### Reframing of items

In this paper, the term ‘reframing’ is used to describe scenarios in which participants reinterpret a word or phrase in order to make an item accessible. For example, *“When I saw walking I just kind of took it as wheeling. … I’ll just say wheeling instead, I don’t mind crossing that off and putting that”* (FG2, SF-36v2); “*It didn’t jump out at me right away, so I kind of like, well I’ll just change it to wheel again”* (FG2, HUI); *“Wheeling is our equivalent to walking”* (FG3, HUI). One participant mentioned that wheeling is the natural description for walking when thinking about mobility-related daily activities: *“When I take my dog for a walk, I just say I’m going to go take my dog for a wheel”* (FG2, HUI). Participants mentioned that wheeling allows them to portray their mobility-related health state in a way that feels closer to their actual experience: *“If it said wheeling I can do it no problem, they can gather that my health is pretty good”* (FG3, SF-36v2).

## Discussion

This study explored diverging item interpretations in the descriptive systems of health state instruments, using the example of individuals with SCI and mobility-related items. Study participants consistently reported difficulties in finding relevant response options for mobility-related items. Consequently, some participants interpreted items differently from the originally intended meaning. Although this allowed them to describe their level of mobility ‘correctly’, from their own perspective, such diverging item interpretations pose a direct threat to the validity of health state instruments.

Narrow wording was a central topic in the focus group discussions. Previous findings have questioned the suitability of the word ‘walking’ for the SCI population, although not within the context of health state instruments. For example, the word ‘walking’ has been considered unacceptable and even perceived as offensive and inappropriate in an investigation of the SF-36 and the Quality of Well-Being scale in an SCI population [21]. Similarly, respondents in a qualitative study reported problems in interpreting the physical functioning items of the SF-36 due to narrow wording [10]. Dijkers concluded that the wording used to capture physical functioning in the SF-36v2 is suitable only if the aim is to capture impairment – but not if the level of functioning or participation is to be measured [22]. Despite the important role of wording for item interpretation (and, therefore, for the validity of health state instruments), it has so far received little attention in the health economics literature.

A number of study participants proposed reframing as a response strategy to provide a more ‘correct’ description of their level of mobility, i.e., choosing to replace “walking” with “wheeling”. Within the context of health state instruments, this means that the value assigned to a particular reframed health state description is incorrect, because participants in the respective valuation study did not value the reframed health state. This is one example of how reframing can pose a threat to the validity of health state instruments. A related issue is that the recipient of the data is unlikely to know if reframing took place, and for whom it took place [10]. When making use of data from questionnaires, a tacit assumption is that the respondents are relatively homogeneous in their item interpretations, or at least that any variance in item interpretation can be handled as normally distributed measurement error. This assumption allows the recipient of the data to disregard respondent variation in interpretation of the scales since it is assumed that the errors will cancel out, on average, given a large sample size. However, if specific groups of respondents systematically deviate in their interpretation of the scales, the results for these groups will be biased, regardless of the sample size. Examining mobility within the context of SCI has highlighted that reframing is problematic and it is highly likely that reframing occurs in other patient groups, e.g., individuals with motor neurone disease [34], and for items/dimensions other than mobility.

A second recurring issue in the focus groups was the way aids and appliances are referred to in health state instruments. Some participants wanted the wheelchair to be mentioned explicitly, while others would have liked to have instructions on if and how aids should be taken into account. These reactions are in line with findings from a study involving patients with motor neurone disease responding to the 3-level EQ-5D (EQ-5D-3L) [34]. Individuals using aids and appliances (e.g., hearing aids, eyeglasses and wheelchairs) or medications (e.g., painkillers) that influence the symptoms and/or functionings described in the descriptive systems could face comparable challenges if no explicit instruction to aid interpretation is provided; as shown in Table 1, there is no consensus among current instruments. Asada developed a classification that differentiates between medical technologies (e.g., medication), nonhuman aids (e.g., wheelchair), human assistance (e.g., help of another person when walking) and accommodating environmental factors (e.g., a barrier-free physical environment), and suggests to include medical technologies and nonhuman aids only in the assessment of health [35]. To some extent, this position is reflected in the current state of play because none of the health state instruments included in this study (15D, AQoL-8D, EQ-5D-5L, HUI, QWB-SA or SF-6D) refer to environmental factors, such as accessibility and the availability of rehabilitation.

Modifying items is one approach to try to make items more accessible to a broader range of clinical contexts. There have been several attempts in the literature to modify items of non-preference-based HRQoL instruments, primarily focusing on the physical functioning dimension of the SF-36 [21, 36, 37]. However, modifying health state instruments is problematic for several reasons. Firstly, comparisons across different patient groups are no longer valid if an instrument includes modified items or instructions for a particular subgroup of respondents. Secondly, psychometric evidence for the *original* instrument cannot be considered valid evidence for the *modified* version of the instrument. Thirdly, as alluded to above, health state values representing preferences for health states of the original descriptive system should not be applied to health state descriptions from the modified descriptive system.

In recent years there has been a call for more patient involvement in instrument development, as most health state instruments were developed with little input from individuals representative of the intended respondents [10, 38, 39]. The current study contributes to this call by exploring variation in item interpretation from the perspective of individuals living with SCI. An example of the value of such an approach was the participants’ suggestion of a broader conceptualization of mobility, one that is not solely restricted to an individual’s ability to walk. Ideally, exploring item interpretations would have been part of the development process of the respective descriptive systems. The findings of this study have implications beyond the measurement of *preference-based* health status because mobility-related items are ubiquitous in generic HRQoL instruments. More specifically, the findings are valuable to researchers during the process of selecting a suitable instrument, when reevaluating existing instruments, or when developing new instruments. The findings could also help explain how variations between the descriptive systems of different preference-based instruments may contribute to poor agreement between their derived health state values [19, 40].

Further qualitative research in this area should explore whether similar inferences (accessibility, reframing, etc.) can be drawn for items and contexts beyond mobility and SCI. As a case in point, a number of studies have shown that individuals with mental health conditions have difficulties describing their health [41–45]. Secondly, important conceptual and normative questions should be revisited in order to judge the relative merits of different health state instruments. For example, with the EQ-5D-5L becoming established as a widely-used health state instrument, should it include explicit instruction as to whether respondents should be considering medical technologies, nonhuman aids and/or human assistance when completing the instrument; and what are the implications of maintaining the *status quo*?

### Strengths and limitations

Having a group of individuals with significant physical impairments share their perceptions of mobility-related items provided an excellent opportunity to address an under-researched area in health economics. This study is a starting point–and a rallying call–for further exploration of item interpretations in other populations. A limitation of the study is that it comprises secondary analysis of focus group data (limitations with respect to the original study are reported elsewhere [19]). Mobility was not the sole focus of the original focus group discussions and, therefore, the structured discussion template was not designed in a way to probe for further clarification or elaboration on mobility-related issues. For example, the questions in the template did not directly encourage participants to discuss ‘item interpretation’ (broadly, questions two and four focused on the applicability and acceptability of the instruments, while questions one and three provided participants with the opportunity to raise any issue related to the instruments). Consequently, it is possible that saturation was not reached with regard to mobility-related issues and, in particular, issues regarding item interpretation. Despite these limitations, the clinical context of the focus group ensured that aspects of mobility were an extensive part of the discussion.

## Conclusions

This study identified four themes that provide insight into the issues individuals living with significant mobility impairment may face when responding to mobility-related items in health state instruments. The observed problems–such as the lack of suitable response options and the reframing of items–pose considerable threats to the validity of health state descriptions, health state values and, ultimately, cost-effectiveness estimates in the evaluation of treatments and interventions. The extent to which other respondent groups experience similar difficulties in interpreting items, whether mobility-related or otherwise, remains unknown.

## Appendix 1

**Table 3:** Wording of mobility-related items in six generic, preference-based health state instruments ^a^

Instrument (item number)	Wording of question/dimension label, the mobility-related item and the respective response options
15D (1)	QUESTION / DIMENSION LABEL: *Mobility* RESPONSE OPTIONS • I am able to walk normally (without difficulty) indoors, outdoors and on stairs. • I am able to walk without difficulty indoors, but outdoors and/or on stairs I have slight difficulties. • I am able to walk without help indoors (with or without an appliance), but outdoors and/or on stairs only with considerable difficulty or with help from others. • I am able to walk indoors only with help from others. • I am completely bed-ridden and unable to move about.
AQoL-8D (3)	QUESTION / DIMENSION LABEL: *Thinking about how easy or difficult it is for you to get around by yourself outside your house (*e.g.*, shopping, visiting):* RESPONSE OPTIONS • Getting around is enjoyable and easy • I have no difficulty getting around outside my house • A little difficulty • Moderate difficulty • A lot of difficulty • I cannot get around unless somebody is there to help me
AQoL-8D (15)	QUESTION / DIMENSION LABEL: *Thinking about your mobility, including using any aids or equipment such as wheelchairs, frames, sticks:* RESPONSE OPTIONS • I am very mobile. • I have no difficulty with mobility. • I have some difficulty with mobility (for example, going uphill). • I have difficulty with mobility. I can go short distance only. • I have a lot of difficulty with mobility. I need someone to help me. • I am bedridden.
EQ-5D-5L (1)	QUESTION / DIMENSION LABEL: *Mobility* RESPONSE OPTIONS • I have no problems in walking about • I have slight problems in walking about • I have moderate problems in walking about • I have severe problems in walking about • I am unable to walk about
HUI (9)	QUESTION / DIMENSION LABEL: *Which one of the following best describes your ability, during the past week, to walk? Note: Walking equipment refers to mechanical supports such as braces, a cane, crutches or a walker.* RESPONSE OPTIONS • Able to walk around the neighbourhood without difficulty, and without walking equipment. • Able to walk around the neighbourhood with difficulty; but did not require walking equipment or the help of another person. • Able to walk around the neighbourhood with walking equipment, but without the help of another person. • Able to walk only short distances with walking equipment, and required a wheelchair to get around the neighbourhood. • Unable to walk alone, even with walking equipment. Able to walk short distances with the help of another person, and required a wheelchair to get around the neighbourhood. • Unable to walk at all.
QWB-SA (7a-7 h (ii))	QUESTION / DIMENSION LABEL: *Over the last three days, did you (please fill in all days that apply):* • Have trouble climbing stairs or inclines or walking off the curb? • Avoid walking, having trouble walking, or walk more slowly than other people of your age? • Limp or use a cane, crutches, or walker? • Avoid or have trouble bending over, stooping or kneeling? • Have any trouble lifting or carrying everyday objects such as books, a briefcase, or groceries? • Have any other limitations in physical movements? • Spend all or most of the day in a bed, chair, or couch because of health reasons? • Spend all or most of the day in a wheelchair? • If in a wheelchair, on which days did someone else control its movement? RESPONSE OPTIONS • No days • Yesterday • 2 days ago • 3 days ago
SF-36v2 (3a-3j)	QUESTION / DIMENSION LABEL: *The following questions are about activities you might do during a typical day. Does your health now limit you in these activities? If so, how much?* • Vigorous activities, such as running, lifting heavy objects, participating in strenuous sports • Moderate activities, such as moving a table, pushing a vacuum cleaner, bowling, or playing golf • Lifting or carrying groceries • Climbing several flights of stairs • Climbing one flight of stairs • Bending, kneeling or stooping • Walking more than a kilometre • Walking several hundred metres • Walking one hundred metres • Bathing or dressing yourself RESPONSE OPTIONS • Yes, limited a lot • Yes, limited a little • No, not limited at all

^a^ The item phrasing provided in this table is for illustration only and does not reflect the formatting in the original instruments

## Appendix 2

**Table 4:** Overview of mobility-related content from the focus group transcripts^a^

Theme	HUI	EQ-5D-5L	15D	AQoL-8D	QWB-SA	SF-36v2	General remark
Wording of mobility	*Wheeling… is our equivalent to walking, and it is so different mechanically* FG3	*You can still get around but you just can’t do it by walking* FG2	*I don’t think mobility is just related to walking* FG1	*I liked the wording of the mobility question, it doesn’t exclude anybody getting around, however you get around on your hands or knee or do cartwheels* FG3		*Can you walk today? Well, no, some people can, of course, but the majority of them can’t, they have trouble getting around. Could you walk a hundred yards well I can wheel a hundred yards today* FG3	
		*The first question is an absurd question* FG1	*But the other one it jumps at you right away the first thing can you walk? It’s like oh poor me, thanks for slapping me in the face with that one* FG2	*How do you get around it seems whatever way you use canes, crutches, wheelchair or you use your legs* FG3		*I just wonder how the answers would be interpreted … like I say the walking one hundred yards, well I can wheel hundred yards without thinking, but I’m not going to put that on here, I’m going to put no, and they look at it and oh that’s a problem* FG3	
Wording of mobility (continued)			*I didn’t like the choice of words…, I don’t think walking is your only parameter for mobility* FG1	*It doesn’t say anything about walking with wheelchairs, it just says mobility* FG2		*So this section I don’t know what they are going to get from that. You’re limited to walking more than a mile, whereas it gives you the option of wheeling right, it’s getting more specific* FG3	
			*Very first question right away can you walk, okay, well thank you very much for that one. I kind of, you know, next one* FG2	*In brackets it says, for example going uphill, that’s where I struggle* FG2		*If it said wheeling I can do it no problem, they can gather that my health is pretty good* FG3	
			*It’s a terrible use of words* FG1				
Reference to aids and appliances	*It didn’t give you the option of wheelchairs* FG3		*Doesn’t even mention the fact of being wheelchair bound* FG1	*This one includes the wheelchair you know* FG1	*Is there any reason why there wasn’t in the, ‘which health aides do you usually use why.’ there isn’t wheelchairs or any disability things there? It’s limited* FG1	*There should be more questions for wheelchairs, you know, spinal cord, because I tell you, you know, you can’t walk* FG3	*I found the questions were I think vague when they came to wheelchairs especially there’s no* FG3
Reference to aids and appliances (continued)	*They’re beating around the bush about equipment but they’re not mentioning you’re unable to walk but are you able to get around. It’s a mobility thing, … [It needs to be] defined a little bit more, if you’re trying to find wheelchair people* FG2		*I think there needs to be more questions applying to people,…do you need a wheelchair to get around?* FG3	*I liked number fifteen when it talks [about] mobility, it also included aids or equipment such as wheelchairs, frames, stations and stuff because if… I have the equipment I feel comfortable with my mobility* FG1	*How would you want someone like a paraplegic to answer those questions? I mean they are not applicable to people who are confined on a wheelchair* FG1	*There should be more questions on wheelchairs right, can you walk a mile or four weeks ago, well da I don’t know, can you wheel a couple of blocks in four weeks, oh yeah, you know, more questions about wheelchairs, it’s very open right?* FG3	*Well for us … if the wheelchair is in there …, I’d feel more part of it* FG1
	*If I’m in a standing frame or a walker I can walk, but I can’t walk unassisted…, none of them really helped me answer that one* FG1		*And there’s no option on wheelchair* FG1	*It doesn’t say anything about walking with wheelchairs, it just says mobility. Just think about your mobility including using any aids or equipment* FG2	*Where does a wheelchair fit in?* FG1	*So this section I don’t know what they are going to get from that. You’re limited to walking more than a mile, whereas it gives you the option of wheeling right, it’s getting more specific. If it said wheeling they can gather my health is pretty good* FG3	
			*Written a little bit like wheelchairs isn't really part of the questionnaire* FG2	*How do you get around it seems whatever way you use canes, crutches, wheelchair or you use your legs* FG3			
Lack of suitable response options	*If I’m in a standing frame or a walker I can walk, but I can’t walk unassisted…, none of them really helped me answer that one* FG1		*It is just like just close our eyes and pick one* FG3	*The question seems appropriate, people like this question because the options are relevant as opposed to walking* FG3	*How would you want someone like a paraplegic to answer those questions? I mean they are not applicable to people who are confined on a wheelchair* FG1	*By having questions like this that have nothing to do with me, so they’re not going to get any information, or they may even get a false sense of my health status by asking questions like that* FG3	
			*I thought that question was kind of hard to fill out. …Well it didn’t really have an option for me* per se *because like there again this question is put in front of somebody like they don’t know whether you’re in a wheelchair or you’re a quadriplegic, paraplegic or if you’re bedridden or you can walk with a cane, right?* FG3			*I just wonder how the answers would be interpreted … like I say the walking one hundred yards, well I can wheel hundred yards without thinking, but I’m not going to put that on here, I’m going to put no, and they look at it and oh that’s a problem* FG3	
Lack of suitable response options (continued)			*Even with help to being bedridden, an invalid basically, like I mean you feel almost insulted that there isn’t any recognition for people that are in between* FG1			*This whole one section here I mean none of these apply basically, walking a mile* etc. FG3	
			*The first question right there I didn’t know which one to tick* FG3			*It turns me off, I mean there is no relevancy to me* FG1	
			*I don’t walk but I’m also not completely bedridden either* FG3				
			*I can’t walk at all but even if someone is trying to help me, I still can’t walk but I’m not in bed either* FG1				
Reframing of items	*It didn’t jump out at me right away, so I kind of like, well I’ll just change it to wheel again* FG2		*Yeah it’s kind of walking to wheel* FG2			*When I saw walking I just kind of took it as wheeling. But the other one jumps at your right away…, I’ll just say wheeling instead, I don’t mind crossing that off and putting that* FG2	
	*When I take my dog for a walk, I just say I’m going to go take my dog for a wheel* FG2					*If it said wheeling I can do it no problem, they can gather that my health is pretty good* FG3	
	*Wheeling is our equivalent to walking* FG3						

^a^ Some quotes were assigned to more than one theme. Abbreviations FG1, FG2 and FG3 indicate the respective focus group transcript for each quote

## Abbreviations

AQoL: Assessment of Quality of Life
AQoL-8D: Assessment of Quality of Life 8-dimension questionnaire
HRQoL: health-related quality of life
HUI: Health Utilities Index
HUI-3: Health Utilities Index Mark 3
QALY: quality-adjusted life year
QWB-SA: Quality of Well-Being Scale Self-Administered
SCI: spinal cord injury
SF-36v2: 36-item Short Form health survey version 2

## References

[1] Neumann PJ, Goldie SJ, Weinstein MC (2000). Preference-based measures in economic evaluation in health care. Annu Rev Public Health.

[2] Patrick D, Deyo R (1989). Generic and disease-specific measures in assessing health status and quality of life. Med Care.

[3] Canadian Agency for Drugs and Technologies in Health (CADTH) (2006). Guidelines for the Economic Evaluation of Health Technologies.

[4] National Institute for Health and Care Excellence (2013). Guide to the Methods of Technology Appraisal 2013.

[5] Dutch National Health Care Institute (2015). Guideline for Conducting Economic Evaluations in Health Care.

[6] Drummond M, Sculpher M, Claxton K (2015). Methods for the Economic Evaluation of Health Care Programmes. Oxford university press.

[7] Hunt S (1997). The problem of quality of life. Qual Life Res.

[8] Richardson J, McKie J, Bariola E, Culyer A (2014). Multiattribute Utility Instruments and Their Use. Encyclopedia of Health Economics.

[9] AER Association (2014). American Psychological Association, National Council on Measurement in Education, Joint Committee on Standards for Educational and Psychological Testing (U.S.): Standards for Educational and Psychological Testing.

[10] Mallinson S (2002). Listening to respondents: a qualitative assessment of the Short-Form 36 Health Status Questionnaire. Soc Sci Med.

[11] Dolan P (2000). Effect of age on health state valuations. J Health Serv Res Policy.

[12] Kharroubi SA (2015). A Comparison of Japan and U.K. SF-6D health-state valuations using a non-parametric Bayesian method. Appl Health Econ Health Policy.

[13] Richardson J, Khan MA, Iezzi A, Maxwell A (2015). Comparing and explaining differences in the magnitude, content, and sensitivity of utilities predicted by the EQ-5D, SF-6D, HUI 3, 15D, QWB, and AQoL-8D multiattribute utility instruments. Med Decis Mak.

[14] Moock J, Kohlmann T (2008). Comparing preference-based quality-of-life measures: results from rehabilitation patients with musculoskeletal, cardiovascular, or psychosomatic disorders. Qual Life Res.

[15] Fryback DG (2010). Comparison of five health-realted quality of life indexes using item response theory anlysis. Med Decis Mak.

[16] Whitehurst DGT, Mittmann N, Noonan VK, Dvorak MF, Bryan S (2016). Health state descriptions, valuations and individuals’ capacity to walk : a comparative evaluation of preference-based instruments in the context of spinal cord injury. Qual Life Res.

[17] Keeley T, Al-Janabi H, Lorgelly P, Coast J (2013). A qualitative assessment of the content validity of the ICECAP-A and EQ-5D-5L and their appropriateness for use in health research. PLoS One.

[18] Matza LS, Boye KS, Stewart KD, Curtis BH, Reaney M, Landrian AS (2015). A qualitative examination of the content validity of the EQ-5D-5L in patients with type 2 diabetes. Health Qual Life Outcomes.

[19] Whitehurst D, Suryaprakash N, Engel L, Mittmann N, Noonan VK, Dvorak MF, Bryan S (2014). Perceptions of individuals living with spinal cord injury toward preference-based quality of life instruments: a qualitative exploration. Health Qual Life Outcomes.

[20] Froehlich-Grobe K, Andresen EM, Caburnay C, White GW (2008). Measuring health-related quality of life for persons with mobility impairments: an enabled version of the short-form 36 (SF-36E). Qual Life Res.

[21] Andresen EM, Fouts BS, Romeis JC, Brownson CA (1999). Performance of health-related quality-of-life instruments in a spinal cord injured population. Arch Phys Med Rehabil.

[22] Dijkers MPJM (2005). Quality of life of individuals with spinal cord injury: A review of conceptualization, measurement, and research findings. J Rehabil Res Dev.

[23] Tourangeau R, Rips L, Rasinski K (2000). The Psychology of Survey Response. Cambridge University Press.

[24] Krosnick JA (1999). Survey research. Annu Rev Psychol.

[25] Schwartz CE, Andresen EM, Nosek MA, Krahn GL (2007). Response shift theory: important implications for measuring quality of life in people with disability. Arch Phys Med Rehabil.

[26] Donovan J, Frankel S, Eyles J (1993). Assessing the need for health status measures. J Epidemiol Community Health.

[27] Sintonen H (2001). The 15D instrument of health-related quality of life: properties and applications. Ann Med.

[28] Richardson J, Iezzi A, Khan MA, Maxwell A (2014). Validity and reliability of the Assessment of Quality of Life (AQoL)-8D multi-attribute utility instrument. Patient.

[29] Herdman M, Gudex C, Lloyd A, Janssen M, Kind P, Parkin D, Bonsel G, Badia X (2011). Development and preliminary testing of the new five-level version of EQ-5D (EQ-5D-5L). Qual Life Res.

[30] Furlong W, Feeny DH, Torrance GW, Barr RD (2001). The Health Utilities Index (HUI®) system for assessing health-related quality of life in clinical studies. Ann Med.

[31] Seiber W, Groessl E, David K, Ganiats T, Kaplan R (2008). Quality of Well Being Self-Administered (QWB-SA) Scale User’s Manual.

[32] Brazier J, Roberts J, Deverill M (2002). The estimation of a preference-based measure of health from the SF-36. J Health Econ.

[33] Ritchie J, Lewis J, Nicholls C, Ormston R (2013). Qualitative Research Practice: A Guide for Social Science Students and Researchers. Sage.

[34] Green C, Kiebert G, Murphy C, Mitchell JD, O’Brien M, Burrell A, Leigh PN (2003). Patients’ health-related quality-of-life and health state values for motor neurone disease/amyotrophic lateral sclerosis. Qual Life Res.

[35] Asada Y (2005). Medical technologies, nonhuman aids, human assistance, and environmental factors in the assessment of health states. Qual Life Res.

[36] Lee BB, Simpson JM, King MT, Haran MJ, Marial O (2009). The SF-36 walk-wheel: a simple modification of the SF-36 physical domain improves its responsiveness for measuring health status change in spinal cord injury. Spinal Cord.

[37] Whitehurst DGT, Engel L, Bryan S (2014). Short Form health surveys and related variants in spinal cord injury research: A systematic review. J Spinal Cord Med.

[38] Stevens K, Palfreyman S (2012). The use of qualitative methods in developing the descriptive systems of preference-based measures of health-related quality of life for use in economic evaluation. Value Health.

[39] Stevens KJ (2016). How well do the generic multi-attribute utility instruments incorporate patient and public views into their descriptive systems?. Patient.

[40] Richardson J, Iezzi A, Khan MA (2015). Why do multi-attribute utility instruments produce different utilities: the relative importance of the descriptive systems, scale and ‘micro-utility’ effects. Qual Life Res.

[41] Connell J, O’Cathain A, Brazier J (2014). Measuring quality of life in mental health: are we asking the right questions?. Soc Sci Med.

[42] Papaioannou D, Brazier J, Parry G (2011). How valid and responsive are generic health status measures, such as EQ-5D and SF-36, in schizophrenia? A systematic review. Value Health.

[43] Brazier J, Connell J, Papaioannou D, Mukuria C, Mulhern B, Peasgood T, Jones ML, Paisley S, O’Cathain A, Barkham M, Knapp M, Byford S, Gilbody S, Parry G (2014). A systematic review, psychometric analysis and qualitative assessment of generic preference-based measures of health in mental health populations and the estimation of mapping functions from widely used specific measures. Health Technol Assess.

[44] Jenkinson C, Peto V, Coulter A (1996). Making sense of ambiguity : evaluation of internal reliability and face validity of the SF 36 questionnaire in women presenting with menorrhagia. Qual Heal Care.

[45] Kessler RC, Aguilar-gaxiola S, Alonso J, Chatterji S, Lee S, Ormel J, Üstün TB, Wang PS (2009). World Mental Health (WMH) Surveys. Epidemiol Psichiatr Soc.

